# Delivery of miR-424-5p via Extracellular Vesicles Promotes the Apoptosis of MDA-MB-231 TNBC Cells in the Tumor Microenvironment

**DOI:** 10.3390/ijms22020844

**Published:** 2021-01-15

**Authors:** Yueyuan Zhou, Yusuke Yamamoto, Fumitaka Takeshita, Tomofumi Yamamoto, Zhongdang Xiao, Takahiro Ochiya

**Affiliations:** 1 State Key Laboratory of Bioelectronics, School of Biological Science and Medical Engineering, Southeast University, Nanjing 210096, China; yueyuanzhou16@hotmail.com; 2 Division of Molecular and Cellular Medicine, National Cancer Center Research Institute, 5-1-1 Tsukiji, Chuo-ku, Tokyo 104-0045, Japan; yuyamamo@ncc.go.jp (Y.Y.); toyamamo@ncc.go.jp (T.Y.); 3 Department of Functional Analysis, FIOC, National Cancer Center Research Institute, 5-1-1 Tsukiji, Chuo-ku, Tokyo 104-0045, Japan; futakesh@ncc.go.jp; 4 Department of Molecular and Cellular Medicine, Institute of Medical Science, Tokyo Medical University, 6-1-1 Shinjuku, Shinjuku-ku, Tokyo 160-8402, Japan

**Keywords:** exosome, miR-424-5p, PD-L1, triple-negative breast cancer, mesenchymal stromal cell

## Abstract

Programmed cell death ligand-1 (PD-L1) overexpressed on cancer cells has emerged as a key inhibitor that maintains the immunosuppressive microenvironment through its interaction with the PD-1 receptor in cancer. Here, we demonstrated that miR-424-5p delivery via extracellular vesicles (EVs) derived from adipose tissue-mesenchymal stromal cells (AT-MSCs) partly promotes proinflammation and enhances antitumor cytotoxicity in vitro and in vivo. Triple negative breast cancer (TNBC) exhibits increased expression of *PD-L1*, and *PD-L1* is positively correlated with the overall survival of patients with TNBC. PD-L1 shows relatively higher expression in MDA-MB-231 (MM231) cells and can be downregulated by miR-424-5p. Furthermore, miR-424-5p transported by EVs can increase the secretion of proinflammatory cytokines, decrease the secretion of anti-inflammatory cytokines and promote the apoptosis of tumor cells. The intratumoral administration of miR-424-5p-EVs significantly slowed tumor growth. In conclusion, these results demonstrate that EVs may serve as a delivery system for novel immunotherapies for TNBC through the miR-424-5p/PD-L1 pathway.

## 1. Introduction

Triple-negative breast cancer (TNBC) comprises 15–20% of all breast cancers and is a more aggressive and destructive subtype with poor prognosis, high rates of relapse and more frequent metastasis to the lung and brain [1]. It is characterized by the lack of expression of the estrogen receptor (ER) and progesterone receptor (PR) and the amplification of human epidermal growth factor receptor 2 (HER2). Therefore, TNBC is unresponsive to targeted therapies. Traditional treatment options of surgery, chemotherapy and radiotherapy offer poor clinical outcomes [2]. Recent studies have revealed that increasing lymphocytic infiltration in ER-/HER2- breast cancer has prognostic value and that programmed death-ligand-1 (PD-L1) upregulation is associated with improved survival in basal tumors [3,4].

PD-L1, a member of the B7 family, is a putative type I transmembrane protein expressed on tumor cells, natural killer cells (NKs) and dendritic cells (DCs). When combined with its ligand PD-1, PD-L1 can induce T cell apoptosis and promote T cell differentiation towards regulatory T cells, thereby leading to tumor immune evasion [5,6]. Blockade of the PD-1/PD-L1 pathway has led to striking results in several solid tumors including melanoma, bladder cancer and non-small cell lung cancer [7,8]. Atezolizumab, the anti-PD-L1 monoclonal antibody, has received the FDA approval in March 2019, based on results from the IMpassion130 trial that showed a significant increase of overall survival (OS) for patients with PD-L1 positivity (PD-L1 IC ≥ 1%), treated with atezolizumab plus nab-paclitaxel versus nab-paclitaxel alone as first line therapy for metastatic TNBC. Additionally, VENTANA PD-L1 (SP142) assay, to determine PD-L1 positivity, was FDA approved by results from the Impassion130 study [9,10]. Recently, pembrolizumab has demonstrated promising results in early-stage TNBC that can lead in the near future to its approval in the (neo) adjuvant setting [11]. It can be assumed that the PD-L1-positive subgroup of TNBC will potentially benefit the most from the use of ICIs, especially as combination therapy.

Extracellular vesicles (EVs), ranging from 30 to 150 nm, are released by all cell types and consist of various types of lipids, proteins and nucleic acids including mRNAs, noncoding RNAs and microRNAs (miRNAs) [12,13]. MiRNAs uploaded in EVs can be transferred and regulate the expression of target genes in recipient cells, which suggests that transfer of miRNAs via EVs is involved in intercellular communication [14]. EVs derived from adipose tissue-mesenchymal stromal cells (AT-MSCs) possess the natural ability to home on to breast tumor sites and have weak immunogenicity with low expression of MHC class I based on their parental cell sources [15,16,17]. Notably, PD-L1 has been identified as the downstream target of several miRNAs, such as miR-34a and miR-873 [18,19]. Therefore, we hypothesized that AT-MSC-EV-mediated delivery of miRNA promotes the apoptosis of TNBC cells in the tumor microenvironment by suppressing PD-L1.

Our previous study analyzed the distinct miRNAs expressed in AT-MSC-exosomes, and PD-L1 was predicted to be a potential target of these miRNAs in silico. In the current study, we focused on the miR-424-5p/PD-L1 axis in TNBC MDA-MB-231 (MM231) cells and the effect of EV-mediated delivery of miR-424-5p in vitro and in vivo. Our findings might provide a novel approach for immune checkpoint blockage via EVs that enhances the immune response against tumor cells in TNBC.

## 2. Results

### 2.1. PD-L1 Expression Is Increased in TNBC

**Figure 1:**
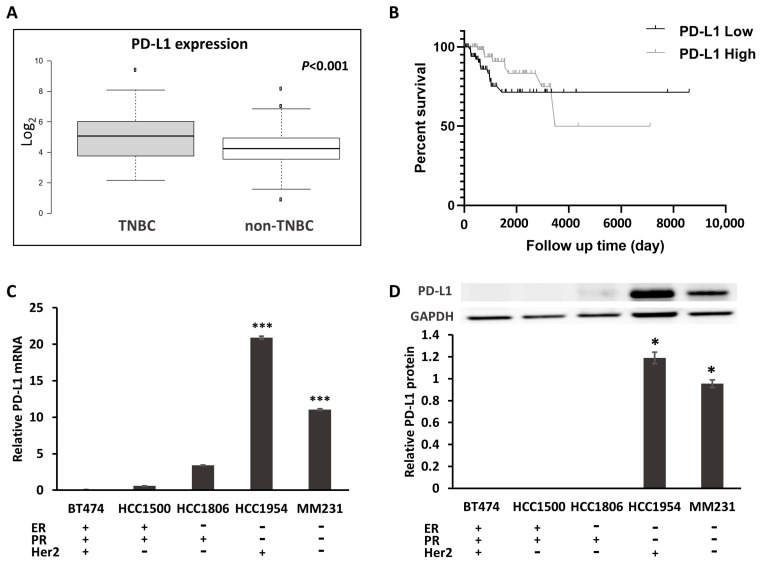
The expression of PD-L1 in TNBC. (**A**) Box plot (from 826 cases of breast cancer downloaded from TCGA) generated using BoxplotR (http://shiny.chemgrid.org/boxplotr/). Center lines show the medians; box limits indicate the 25th and 75th percentiles as determined by R software; whiskers extend 1.5 times the interquartile range from the 25th and 75th percentile, outliers are represented by dots. *n* = 214, 612 sample points. (**B**) Kaplan–Meier overall survival analysis (from 214 TNBC cases of breast cancer downloaded from TCGA). *p* = 0.0329. (**C**) mRNA expression of *PD-L1* in breast cancer cell lines. (**D**) The protein expression of PD-L1, compared with the normal breast epithelial MCF-10A cell line. * *p* < 0.05, *** *p* < 0.001.

To evaluate the presence of PD-L1 in breast cancer, we analyzed TCGA RNA sequencing data to determine whether the *PD-L1* transcript exists. Differential *PD-L1* expression was observed, with significantly higher expression in TNBC (*n* = 214) than in non-TNBC (*n* = 612) (*p* < 0.001; Figure 1A). To further examine the association of *PD-L1* expression with clinical features, a Kaplan–Meier analysis was conducted to determine whether the expression of *PD-L1* in tumors was associated with overall survival. *PD-L1* expression was used to assign patients to the high (above the median value 4.95) or low (lower than 4.95) expression groups.

The patients with TNBC who expressed high levels of *PD-L1* had a longer overall survival (OS) (*p* = 0.03) (Figure 1B). We found significant correlations between *PD-L1* expression and clinical characteristics, including patient age (*p* = 0.027) and status (*p* = 0.038), in TNBC (Supplementary Table S1A). However, there were no significant correlations between *PD-L1* expression and grade (*p* = 0.317) in TNBC, or between patient age (*p* = 0.208), grade (*p* = 0.140) and status (*p* = 0.425) in non-TNBC (Supplementary Table S1A,B). To assess the PD-L1 expression in vitro, we next compared PD-L1 expression in the human mammary epithelial cell line MCF10A and various human breast cancer cell lines. As shown in Figure 1C, *PD-L1* exhibited increased mRNA expression in HER2+ HCC1954 and TNBC MM231 cells compared with that in MCF10A and other types of breast cancer cell lines through qRT-PCR. Western blotting also confirmed the relatively high expression of PD-L1 protein in HCC1954 and MM231 cells (Figure 1D).

### 2.2. PD-L1 Is Directly Regulated by miR-424-5p

**Figure 2:**
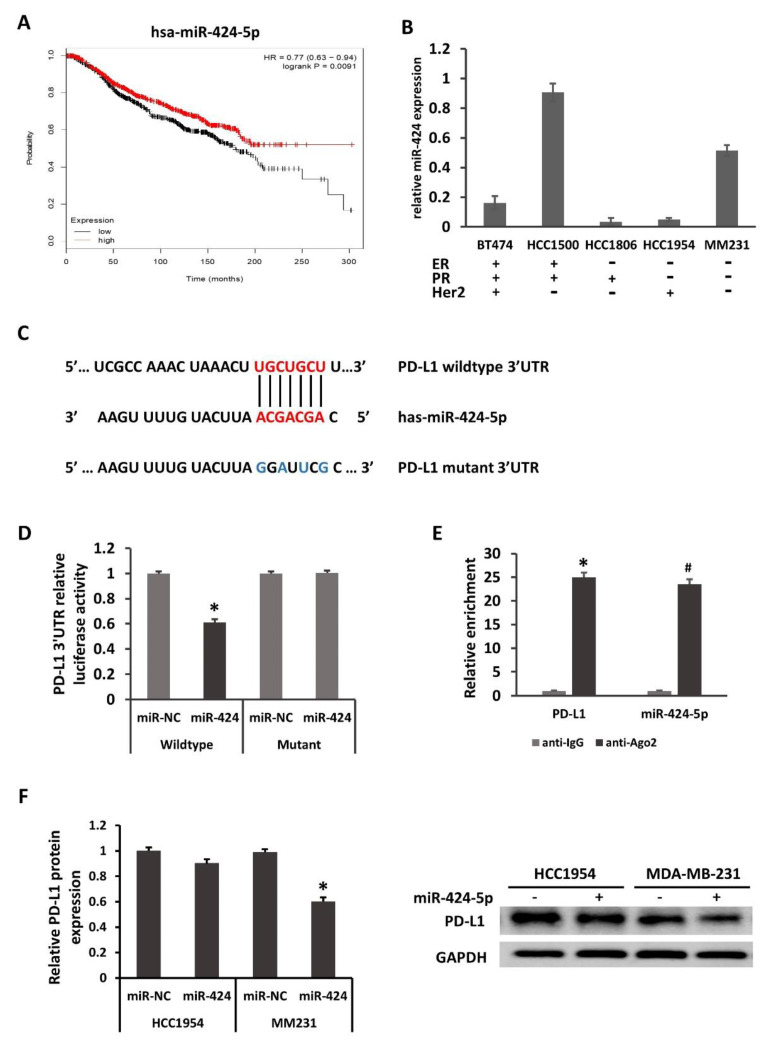
PD-L1 is directly regulated by miR-424-5p. (**A**) Kaplan–Meier overall survival analysis (from 1262 cases of breast cancer, kmplot.com, ID in KMPLOT: hsa-miR-424) revealed that patients with high miR-424-5p expression exhibited longer overall survival times. *p* = 0.0091. miR, microRNA; HR, hazard ratio. (**B**) miR-424-5p expression in several breast cancer lines, compared with the normal breast epithelial MCF-10A cell line. (**C**) PD-L1 is a potential target of miR-424. The binding sites in the 3′UTR of *PD-L1* are shown. (**D**) Luciferase activity from cotransfection of wild-type (WT) and mutant (Mut) *PD-L1* regions with miR-424 or negative control (miR-NC) in HEK293T cells. (**E**) RNA from MM231 cells precipitated with anti-Ago2 or nonspecific rabbit IgG were analyzed via qRT-PCR assay. Relative quantification for *PD-L1* transcript was performed using GAPDH mRNA as a reference gene. The relative expression of PD-L1 protein in HCC1954 and MM231 (**F**) were determined by Western blotting 48 h after transfection. Relative quantification for PD-L1 protein was performed using GAPDH protein as a reference protein. * *p* < 0.05, ^#^ *p* < 0.05.

According to our previous study, *PD-L1* was one of the target genes of miR-424-5p. Kaplan–Meier survival analysis revealed that patients with increased miR-424-5p expression exhibited longer overall survival times, suggesting that miR-424-5p acted as a tumor suppressor (Figure 2A). As shown in Figure 2B, miR-424-5p expression was relatively lower in breast cancer cell lines than that in MCF10A. Although the expression of miR-424-5p in all breast cancer cell lines was not strictly inversely correlated with that of *PD-L1* mRNA, these results suggested that miR-424-5p may serve as a tumor suppressor in breast cancer. It was bioinformatically predicted that PD-L1 is a potential target of miR-424-5p using the public database TargetScan. The Figure 2C illustrated the corresponding sequences of the miR-424-5p, wildtype *PD-L1* 3′UTR and mutation of *PD-L1* 3′UTR. Luciferase reporter assays were performed with the wild-type *PD-L1* mRNA 3′-UTR and the mutant with changes in the miR-424 -5p binding site in the HEK293T cell line. As shown in Figure 2D, luciferase activity was suppressed only in cells cotransfected with the wild-type *PD-L1* mRNA 3′-UTR and miR-424-5p mimics but not in cells transfected with the mutants and the miRNA negative control (miR-NC), suggesting that miR-424-5p targets *PD-L1* through the 3′-UTR. Our AGO2-RNA RIP results displayed that both *PD-L1* and miR-424-5p were highly enriched by anti-Ago2 when compared with the IgG antibody, hinting the endogenous interaction between PD-L1 and miR-424-5p expression (Figure 2E). Collectively, it was confirmed that miR-424-5p directly targeted PD-L1.

Further transfection was carried out in HCC1954 and MM231 cells. At 48 h after transfection, it was subsequently demonstrated by Western blotting that miR-424-5p overexpression significantly downregulated the protein level of PD-L1 in MM231 cells; however, miR-424-5p only slightly suppressed PD-L1 protein expression in HCC1954 cells (Figure 2F). The effect of miRNA regulation could be diluted by target abundance, which means that regulation efficiency varies with cell types. The decay rates of mRNA also affected the RNAi efficiency. These factors may help us understand the differentiation of PD-L1 downregulation in HCC1954 and MDA-MB-231 by miR-424-5p. Taken together, these findings indicate that miR-424-5p mediates the suppression of PD-L1 in MM231 cells by binding to the 3′-UTR.

### 2.3. EVs Are Secreted into AT-MSC Culture Supernatant

According to our previous work, miR-424-5p is specifically expressed in EVs derived from AT-MSCs compared to EVs derived from MSCs isolated from bone marrow, Wharton’s jelly and human exfoliated deciduous teeth [20]. To increase the efficiency of miR-424-5p delivery, we increased the expression of miR-424-5p in EVs through transfecting AT-MSCs with miR-424-5p. EVs were collected from AT-MSCs transfected with miR-424-5p mimics.

**Figure 3:**
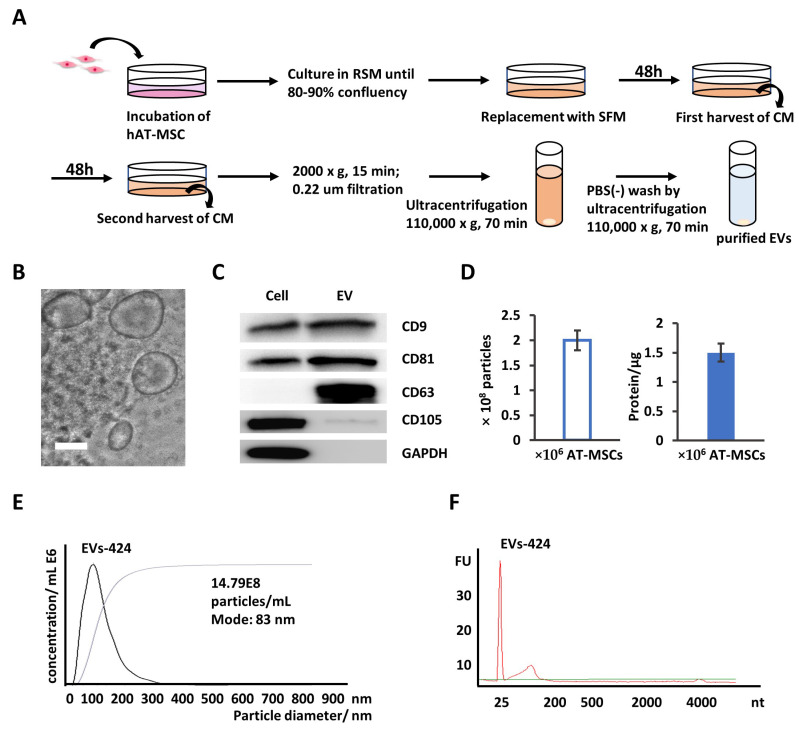
Isolation and characterization of extracellular vesicles (EVs) derived from adipose tissue-mesenchymal stromal cells (AT-MSCs). (**A**) EVs were isolated from the conditioned medium of AT-MSCs following the multicentrifugation method. (**B**) A phase-contrast transmission electron micrograph of the isolated exosomes. Scale bar: 100 nm. (**C**) Protein from AT-MSC cell lysates and EVs was analyzed by Western blotting with antibodies against CD9, CD63, CD81, CD105 and GAPDH. (**D**) Yields of EVs derived from AT-MSCs. Protein amounts and particle numbers of harvested EVs were determined by the Bradford method and nanoparticle tracking analysis (NTA), respectively. (**E**) Particle size distributions of EVs-424 were measured by NTA and showed a peak at 83 nm. (**F**) Bioanalyzer electropherogram of RNA extracted from EVs-424. The *x*-axis indicates the length of the RNA in nucleotides (nt), and the *y*-axis indicates the fluorescence intensity in arbitrary units. The lowest peak at 25 nt indicates the lower size marker. FU, fluorescent units.

EVs were isolated from the supernatant of AT-MSCs by multistep differential centrifugation (Figure 3A), as previously described [21,22]. To characterize the isolated EVs, it was easily observed that EVs were membrane-bound structures with sizes ranging from 60 to 200 nm using phase-contrast transmission electron microscopy (Figure 3B). Moreover, several reliable marker proteins, including CD9, CD63 and CD81, were present in the EV fraction via Western blotting (Figure 3C), which was consistent with other reports about EVs [23,24]. CD105, the differentiation marker protein and GAPDH were expressed in AT-MSC cell lysate, but not in exosomes [25]. The exosome yields per 10^6^ AT-MSCs per day was approximately 2 × 10^8^ particles, as determined by nanoparticle tracking analysis (NTA), or 1.5 µg protein, as measured by the Bradford method (Figure 3D). The size distribution of the collected EVs was physically homogeneous, with a peak at 80–100 nm according to NTA (Figure 3E). As shown in Figure 3F, RNA from both types of EVs had short RNA size profiles below 300 through the analysis of total RNA. These results indicate that EVs were isolated successfully with this protocol from the culture media of AT-MSCs.

### 2.4. EVs-Mediated Delivery of miR-424 Decreases PD-L1 Expression in Recipient Cells

**Figure 4:**
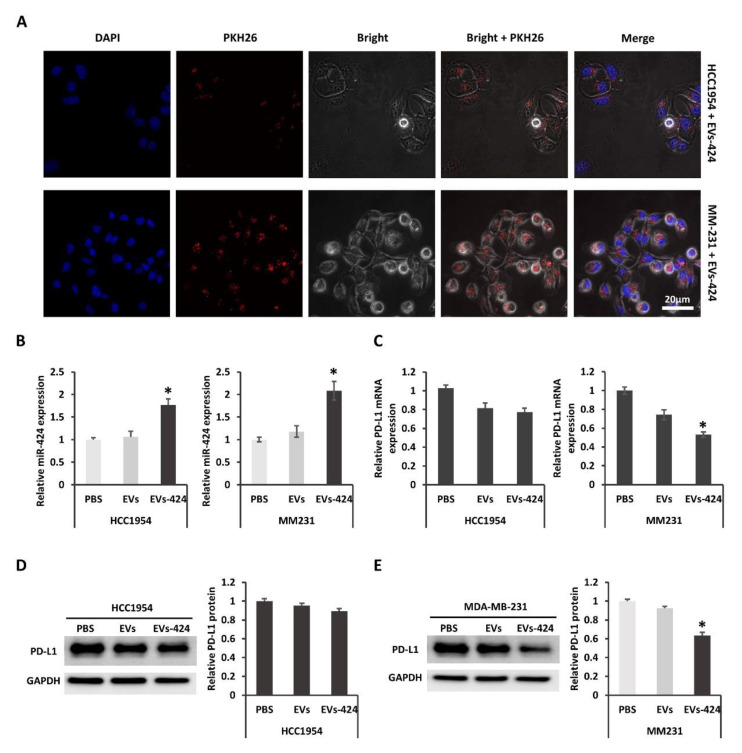
PD-L1 was downregulated by miR-424-5p delivered via EVs. (**A**) The internalization of EVs into MM231 (upper) and HCC1954 (bottom) cells 24 h after incubation. Scale bar: 20 µm. (**B**) Relative expression of miR-424-5p in recipient HCC1954 and MM231 were determined via qRT-PCR assay 24 h after EV incubation. (**C**) Relative mRNA expression of *PD-L1* in HCC1954 and MM231 cells was determined via qRT-PCR assay 24 h after EV incubation. PD-L1 protein expression in HCC1954 (**D**) and MM231 cells (**E**) was compared by Western blotting 48 h after treatment. * *p* < 0.05.

As described above, EVs were collected from the transfected parental AT-MSCs. To examine whether EVs could transfer miR-424 to recipient cells, we firstly check whether the EVs could be uptaken by recipient cells. When EVs-424 cells were incubated with HCC1954 or MM231 cells labeling with PKH26, internalization into the recipient cells was observed by laser scanning confocal microscopy (Figure 4A). After 24 h of EV incubation, the cytoplasm near the nuclei was positive for PKH26 fluorescence, suggesting that the EVs were effectively internalized by the recipient cells. These results were consistent with our previous observation [26]. Moreover, it was confirmed that miR-424-5p expression in HCC1954 and MM231 cells was upregulated after EV incubation as measured by qRT-PCR (Figure 4B). As shown in Figure 4C, the expression of *PD-L1* mRNA in HCC1954 and MM231 cells was decreased after Exo-424 incubation, suggesting that the miR-424 packaged into Exo-424 was functionally transferred into recipient cells, where it modulated target mRNA expression. The downregulation of *PD-L1* mRNA in MM231 was approximately 45%, whereas that in HCC1954 was about 23%. The suppression of *PD-L1* mRNA was stronger in MM231 cells. It was demonstrated that exogenous miRNAs compete with endogenous miRNAs for the protein complex needed for miRNA regulation [27]. Additionally, the effect of miRNA regulation could be diluted by target abundance, which means that the target gene is less downregulated when the miRNA has many highly expressed target genes compared with a few lowly expressed target genes [28]. The decay rates of mRNA vary highly, with half-lives ranging from minutes to days [29]. It was also suggested that short-lived transcripts could be more difficult to perturb using miRNAs and siRNAs [30]. Taken together, it is important to take confounding factors such as competition, target gene expression and mRNA life cycles into account in RNAi efficiency. Furthermore, Western blotting revealed that the expression of PD-L1 protein was decreased significantly in MM231 cells but only slightly in HCC1954 cells after EVs-424 incubation (Figure 4D,E). EVs as a delivery system could constantly release functional miR after uptake. This long-circulating effect achieved sustained downregulation of the target PD-L1 protein.

### 2.5. miR-424-5p Regulates Cytokine Secretion

The cytokine secretion of tumor and immune cells may reflect the immune responses against tumors. The PD-L1/PD-1 axis has been reported to negatively regulate T cell receptor-mediated proliferation and the production of anti-inflammatory cytokines [31]. The downregulation of PD-L1 in HCC1954 cells by miR-424-5p overexpression was not significant. Then we decided to assess whether the upregulation of miR-424-5p affected the tumor-reactive immune response in a coculture system of MM231 cells and peripheral blood mononuclear cells (PBMCs).

**Figure 5:**
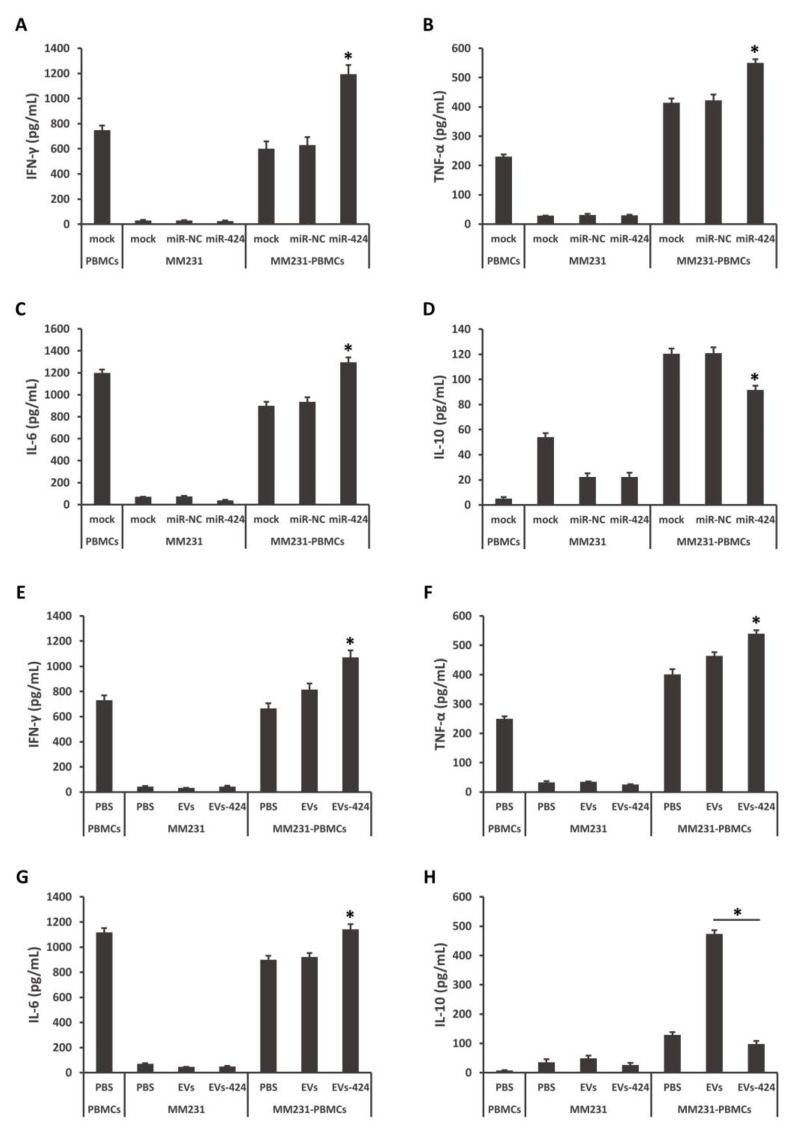
miR-424-5p regulates cytokine secretion in coculture model of MM231/peripheral blood mononuclear cells (PBMCs). MM231 cells were transfected with miR-424-5p mimics (**A**–**D**) or incubated with EVs-424 or EVs (**E**–**H**), and then cocultured with stimulated PBMCs for 16 h. The coculture medium was analyzed for IFN-γ (**A**,**E**), TNF-α (**B**,**F**), IL-6 (**C**,**G**) and IL-10 (**D**,**H**) by ELISA. * *p* < 0.05.

As shown in Figure 5A–D, MM231 cells were pretransfected with miR-424-5p mimics, and then cocultured with stimulated PBMCs. Compared to the control groups, the amounts of inflammatory IFN-γ, TNF-α and IL-6 were increased, while the amounts of anti-inflammatory IL-10 was decreased in the medium. MM231 cells were exposed to EVs-424, and then cocultured with stimulated PBMCs. The contents of IFN-γ, TNF-α and IL-6 were increased and IL-10 was decreased (Figure 5E–H). In particular, EVs-424 could strongly decrease the production of the immunosuppressive cytokine IL-10. IL-10 has been revealed to inhibit CD28 tyrosine phosphorylation, suppress T-cell proliferation and induce anergy in T cells [32,33]. These findings suggest that the restoration of miR-424-5p modulates cytokine production during the interaction of MM231 TNBC cells and immune cells, and that miR-424-5p can be efficiently transferred and exert its effects via EVs.

### 2.6. miR-424-5p Upregulation Promotes Cytotoxicity against Tumors

**Figure 6:**
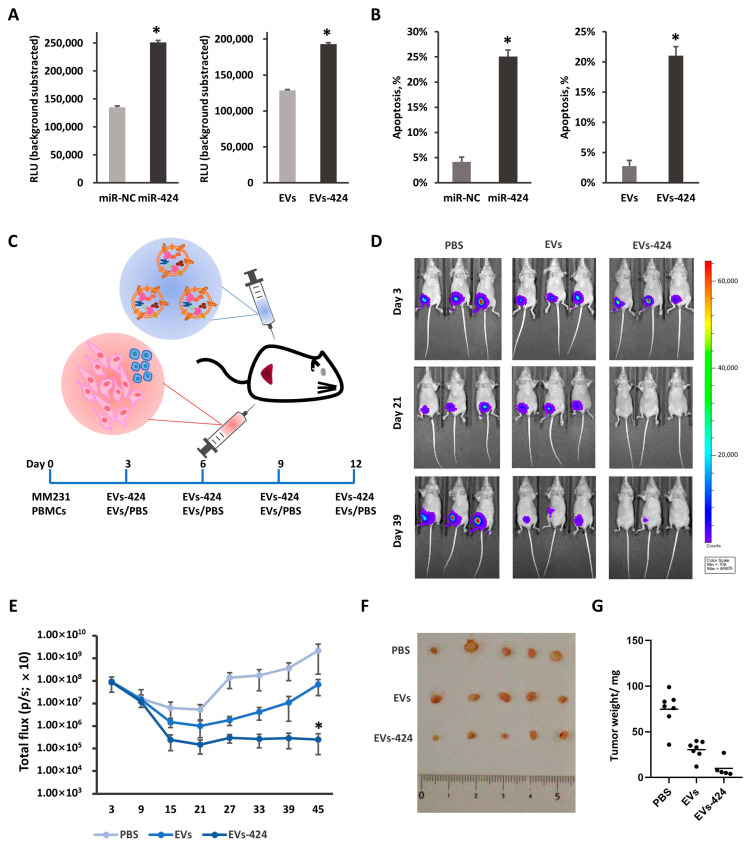
miR-424-5p delivered by EVs enhanced cytotoxicity against tumors. MM-231 cells were transfected with miR-424-5p mimics or incubated with EVs-424, and cocultured with stimulated T cells for 16 h. Caspase 3/7 activities in the coculture system were assessed by a fluorometric assay (**A**). The apoptosis of tumor cells was measured by the LDH release assay (**B**). (**C**) Schematic diagram of animal model. MM231 (2 × 10^6^) and stimulated PBMCs (1 × 10^6^) were injected with Matrigel (100 µL) into the fat pad of BALB/c nude mouse. After 72 h, 30 µg of EVs-424 in 100 µL of PBS was injected intratumorally. EVs were injected 4 times every 72 h. (**D**) Representative bioluminescence images of tumor-bearing mice measured at day 3 (upper), day 21 (middle) and day 39 (bottom). (**E**) Total bioluminescent signal (p/s) for each group at days 3, 9, 15, 21, 27, 33, 39 and 45. (**F**) Tumors dissected from mice of each group were weighed. (**G**) Representative images of the tumor-bearing mice and removed tumors. * *p* < 0.05.

Based on the immunosuppressive role of PD-L1 and the downregulation of PD-L1 by miR-424-5p, it was further investigated whether miR-424-5p upregulation could promote the cytotoxicity of T cells against tumor cells. In the coculture model of tumor cells and T cells, we detected the apoptosis of tumor cells through two types of methods, a Caspase-Glo 3/7 assay and LDH release assay. Caspase 3, a member of the caspase family, is indispensable for apoptotic chromatin condensation and DNA fragmentation in all cell types that have been examined [34,35]. The luminescence signals of caspase 3/7 in the coculture model of MM231/T cells were shown to increase after transfection with miR-424-5p, and EVs-424 treatment, which suggests that the apoptosis was enhanced after miR-424-5p overexpression (Figure 6A).

Then, we assessed extracellular lactate dehydrogenase (LDH) release in the coculture model by measuring the absorbance at 490 nm after coculture. LDH is a soluble cytoplasmic enzyme that is present in almost all cells and is released into the extracellular space when the plasma membrane is damaged [36,37]. As shown in Figure 6B, the lysis ratios of MM231 cells were higher when miR-424-5p was pretransfected. Compared to the control group, pretreatment with Exo-424 similarly increased the lysis ratio. Taken together, these data further show that miR-424-5p overexpression promotes the apoptosis of MM231 cells in coculture models.

To validate the clinical relevance of the EV-mediated delivery of miR-424-5p in vivo, we subcutaneously transplanted MDA-MB-231-D3H2LN cells along with stimulated PBMCs into BALB/c mice, followed by the intratumoral administration of EVs-424 (Figure 6C). Bioluminescent imaging measurements indicated that the tumors in the group treated with EVs-424 grew much more slowly than those in the EVs-treated and PBS-treated groups (Figure 6D). After four EV injections, the growth of the tumors of the EVs-424 group was strongly suppressed compared to that of the tumors of the EVs group and the PBS group (Figure 6D,E). The tumor tissues removed from the EVs-424 group after sacrifice showed a significantly decreased tumor volume (Figure 6F,G). Collectively, the EV-mediated delivery of miR-424-5p exerted antitumor effects in vivo.

## 3. Discussion

TNBC is considered to be more aggressive and results in a poorer prognosis compared to other subtypes of breast cancer. Recently, it has been revealed that TNBC is the immune-modulatory subtype as TNBC is more likely to show increased expression of PD-L1 in the tumor microenvironment, and the presence of tumor infiltrating lymphocytes (TILs) has shown a clear association with clinical outcomes and improved survival [38,39,40]. Therefore, novel immunotherapies, including immune checkpoint inhibitors, have emerged as a promising therapeutic approach for TNBC and metastatic disease. Thus, we consider it urgent to find new approaches that can complement immunotherapy for TNBC.

miRNAs affect the posttranscriptional modulation of protein expression. By using miRNAs to target immune checkpoint molecules, their therapeutic effects can likely be mimicked. It was previously demonstrated by members of our laboratory that PD-L1 is controlled by the miR-197/STAT3/CKS1B network in non-small-cell lung cancer [41]. miR-424 is involved in the chemoresistance of ovarian cancer through PD-L1 signaling [42]. In this study, PD-L1 was identified as a direct target of miR-424-5p via bioinformatic analysis and luciferase reporter assays. TNBC was more frequently associated with the increased expression of PD-L1. Moreover, PD-L1 expression was inversely correlated with miR-424-5p expression. The increased expression of miR-424-5p indicated a longer overall survival time for patients with TNBC.

Due to the elementary role of miRNAs, miRNA delivery may offer clinical benefits when combined with other treatments. Viral delivery systems based on retroviruses, lentiviruses and adenoviruses provide high transfection or infection efficiency, but also have weaknesses, such as toxicity and immunogenicity. In contrast, non-viral carriers modified with lipids and polymers are much safer but have lower transfer efficiencies. EVs that exist in biological fluids possess several advantages, including low immunogenicity, good biocompatibility, low toxicity and ease of modification, making them appropriate choices as delivery vehicles in vivo. Recent studies have indicated that MSCs exhibit the mass production of exosomes and genetically confer to exosomes the natural abilities of homing to tumors and immunoregulation by encapsulating proteins, nucleic acids and other bioactive cargoes [43,44]. It is believed that the migration of MSCs towards the surrounding tumor stroma following chemoattractants in the extracellular matrix and local factors, such as hypoxia and the cytokine environment [45,46]. It has been shown that the breast resident AT-MSCs are actively attracted to the proximity of tumor foci and express both pro- and antitumorigenic genes and miRNAs [47]. Other studies reported that MSCs migrate to hepatic carcinoma [48], glioma [49] and premetastatic niches [50]. Herein, exosomes were collected from miR-424-5p-modified AT-MSCs and served as miR-424-5p delivery systems in vitro and in vivo. These exosomes were internalized into recipient HER2+ HCC1954 and TNBC MM231 cells, after which miR-424-5p expression in recipient cells was upregulated, however the expression of the target PD-L1 protein was significantly suppressed in MM231 cells. Post-transcriptional regulation by miRNAs depends not only on characteristics of individual binding sites in target mRNA molecules, but also on system-level properties such as overall molecular concentrations. Endogenous miRNAs may have their effects diluted by highly abundant target transcripts in particular cell types or states. The differential regulation may result from the involvement of specific miRNAs in universe signaling pathways in different types of cells.

The PD-L1 signaling blockade can induce a sustained immune response in cancer. Cytokines play a critical role in mediating communication in the immune system. IFN-γ released by cytotoxic lymphocytes was revealed to enhance cytotoxic function and cell motility [51,52]. Our work showed that miR-424-5p upregulation promoted the inflammatory response via the increased secretion of the inflammatory cytokines, IFN-γ, TNF-α and IL-6 and decreased the production of the strongly immunosuppressive cytokine IL-10. IL-10 is an anti-inflammatory cytokine that strongly suppresses the function of macrophages and dendritic cells, leading to Th1 differentiation [53]. The effects of miR-424-5p were stronger than EVs-424; however, the effects were also correlated with quantities of exosomes. Modification of miRNA with exosomes-based delivery systems protects the nucleotides from degradation by serum ribonucleases, thereby extending the half-life of oligonucleotides in serum. The apoptosis of MM231 cells was promoted in vitro after incubation with functional EVs-424 based on caspase 3/7 activity and the release of extracellular lactate dehydrogenase. In vivo results revealed that EVs-424 strikingly slowed the growth rates of tumor tissues. During the first three weeks after transplantation, the bioluminescence signals in the PBS-treated and EVs-treated groups presented a weakened tendency. We speculate that the mixed, activated PBMCs had cytotoxic effects on tumor cells, thereby suppressing tumor formation of the remaining MM231 cells. Combined with the results of cytokine secretion, the injected EVs-424 probably facilitates the inflammatory microenvironment by enhancing the secretion of IFN-γ, TNF-α and IL-6 and discouraging the production of immunosuppressive IL-10. Additionally, EVs-424 suppressed the expression of PD-L1, subsequently strengthening the function of effector T cells. Approximately 4 weeks after inoculation of PBMCs and MM231 cells, tumors seemed to recover from inhibitory effects, and the speed of tumor growth rose. Then, the bioluminescence signals and tumor volumes in the EVs-424 group were slower than those in the control groups. These findings suggested that the blockade of PD-L1/PD-1 was effective in delaying the cancer process; however, the combination therapy of immune checkpoint blockade with other treatments, such as traditional chemotherapy or radiotherapy may be a better choice.

In conclusion, EV-mediated delivery of miR-424-5p suppressed PD-L1 signaling, induced an inflammatory microenvironment in TNBC, enhanced the apoptosis of tumor cells in vitro, and inhibited tumor growth in vivo. Our results provide a novel approach for immune checkpoint blockade to improve immunotherapy for TNBC.

## 4. Materials and Methods

### 4.1. Cell Lines and Culture

The MCF10A, BT474, HCC1500, HCC1806, HCC1954 and MM231 cell lines were obtained from the American Type Culture Collection (ATCC). MCF10A cells were cultured in MEBM mammary epithelial cell growth basal medium supplemented with a SingleQuots kit (Lonza, Lonza Group Ltd, Basel, Switzerland). Other breast cancer cell lines were grown under the culture conditions recommended by the manufacturer; and cultured in RPMI 1640 medium supplemented with 10% heat-inactivated FBS (Invitrogen, Thermo Fisher Scientific, Waltham, MA, USA) and antibiotic–antimycotic agents at 37 °C in 5% CO_2_. AT-MSCs were purchased from Invitrogen. AT-MSCs were routinely cultured in reduced serum (2%) medium (MesenPRO RS, Invitrogen, Thermo Fisher Scientific, Waltham, MA, USA) containing 1 × Glutamax (Invitrogen, Thermo Fisher Scientific, Waltham, MA, USA) and 1 × antibiotic antimycotic solution (Invitrogen, Thermo Fisher Scientific, Waltham, MA, USA). All AT-MSCs used were obtained before the eighth passage. PBMCs were obtained by M&S Instruments Inc. (Tokyo, Japan), and all vials were obtained from the same donor (Sample ID#20110518). The PBMCs were cultured in RPMI-1640 medium supplemented with 10% heat-inactivated FBS (Invitrogen, Thermo Fisher Scientific, Waltham, MA, USA), 1% antibiotic–antimycotic agents and 100 ng/mL IL-2 (PEPROTECH, PeproTech Inc., Cranbury, NJ, USA) at 37 °C in 5% CO_2_, and they were activated with CD3/CD28 according to the manufacturer’s instructions (STEMCELL, STEMCELL Technologies Inc., Vancouver, BC, Canada).

### 4.2. Preparation of EVs

Prior to the culture medium collection, the AT-MSCs were washed twice with PBS, and the medium was replaced with fresh serum-free medium (StemPRO SFM, Invitrogen, Thermo Fisher Scientific, Waltham, MA, USA). After incubation for 2–3 days, the medium was collected and centrifuged at 2000× *g* for 15 min at room temperature. The cells were supplemented with fresh SFM and cultured for 2–3 more days, and then the medium was collected and centrifuged as described above. The harvested medium was combined into 1 batch. To thoroughly remove the cellular debris, the supernatant was filtered with a 0.22 μm filter unit (Millipore, Merck Sigma, MA, USA). Then, the CM was ultracentrifuged at 110,000× *g* for 70 min at 4 °C. The pellets were washed with 11 mL of PBS, and after ultracentrifugation, they were resuspended in PBS. EVs-424 was collected from AT-MSCs transfected with miR-424-5p mimics. The exosome fraction of the protein content was assessed by the Bradford method.

### 4.3. Electron Microscopy

The isolated EVs were visualized using a phase-contrast transmission electron microscope (Terabase Inc., Okazaki, Japan) that can generate high-contrast images of the nanostructures of biological materials such as liposomes, viruses, bacteria and cells, without staining procedures that may damage the samples. The natural structure of the sample distributed in solution could be observed by preparing the sample using a rapid vitreous ice-embedding method and cryo-phase-contrast transmission electron microscopy.

### 4.4. Measurement of the Size Distribution and Particle Number by NTA

The isolated EVs were resuspended in PBS at a concentration of approximately 500 µg protein/mL and further diluted 100-fold for nanoparticle tracking analysis (NTA) using a NanoSight system (NanoSight NS3000, Malvern Panalytical, UK). The system utilized the properties of both light scattering and Brownian motion. When the particles in suspension were in the path of the laser beam and scatter light, they could be easily visualized at 20 × magnification with a microscope and recorded by a camera. The camera recorded a 60 s video for further analysis. The particles were tracked individually, and their hydrodynamic diameters were calculated by the Stokes–Einstein equation.

### 4.5. Real-Time Reverse Transcription-PCR

Total RNA and miRNA were isolated from cells and EVs using the RNeasy Mini Kit (QIAGEN, QIAGEN Sciences, MD, USA), and cDNA was produced using the High-Capacity cDNA Reverse Transcription Kit (Applied Biosystems, Thermo Fisher Scientific, Waltham, MA, USA) or the TaqMan MicroRNA Reverse Transcript Kit (Applied Biosystems, Thermo Fisher Scientific, Waltham, MA, USA) according to the manufacturer’s protocols. The TaqMan probes were obtained from Applied Biosystems. The cDNA samples were subjected to real-time PCR using ExTaq Premix (Takara) with a StepOne Real-Time PCR System (Applied Biosciences, Thermo Fisher Scientific, Waltham, MA, USA). The data were collected and analyzed using StepOne Software v2.3 (Applied Biosciences, Thermo Fisher Scientific, Waltham, MA, USA). All mRNA and miRNA quantification data from the cultured cells were normalized according to the expression of glyceraldehyde 3-phosphate dehydrogenase (GAPDH, Hs02758991-g1) and U6 (RUN6B, ID: 001093), respectively.

Primers: PD-L1, Hs01125301_m1 CD274, Cat. 4331182, TaqMan. Has-miR-424-5p, ID: 000604, Cat. 4427975, TaqMan.

### 4.6. Cell Transfection

The transfection of cells was performed with the lipofectamine RNAiMAX reagent (Thermo Fisher Scientific, Waltham, MA, USA) according to the manufacturer’s instructions. Briefly, the cells were seeded with 80% confluence the day before transfection. For the transfection of the miRNA mimics, miR-424 or miR-negative control (miR-NC) mimics (Ambion, Austin, TX, USA) were used for each transfection at a final concentration of 60 nM. After 24 h and 48 h of incubation, the mRNA and protein expression levels were examined by qRT-PCR and Western blotting, respectively, as described below.

### 4.7. Dual-Luciferase Reporter Assay

The PD-L1 3′-UTR regions that contained the predicted miR-424-5p-binding sites and mutated binding sites were amplified by PCR from genomic DNA and inserted into siCHECK vectors (Promega). HEK293T cells (1 × 10^4^) were seeded into a 96-well plate and cotransfected with miR-424-5p or miR-NC mimics and luciferase reporter vectors using the lipofectamine 2000 reagent (Invitrogen, Thermo Fisher Scientific, Waltham, MA, USA). The luciferase activity was quantified 48 h after transfection using the dual-luciferase report assay system (Promega, Promega Corporation, Madison, WS, USA) according to the manufacturer’s instructions. All experiments were repeated at least three times.

Wildtype primers:

Forward5′-AACTTGCTGCTTAATGATTTGCTCACATCTAGT-3′

Reverse 5′- ATTACTACTACAGTTTATGGGGGCGACAAAATT-3′

Mutant primers:

Forward 5′- AACTGTAGTAGTAATGATTTGCTCACATCTAGT-3′

Reverse 5′- ATTACTACTACAGTTTATGGGGGCGACAAAATT-3′

### 4.8. AGO2-RNA Immunoprecipitation

Association of PD-L1 mRNA with the RISC was evaluated by the AGO2-RIP assay using Magna RIPTM RNA-Binding Protein Immunoprecipitation Kit (Millipore, Merck Sigma, MA, USA) and anti-Argonaute2 (anti Ago2) (ab32381, Abcam, Cambridge, UK) or anti-IgG antibody (ab133470, Abcam, Cambridge, UK). The transfected MM231 cell lysates were incubated with A/G magnetic beads together with anti-Ago2 or anti-IgG antibody. Following the removal of protein using proteinase K, the enrichments of PD-L1 and miR-424-5p in immunoprecipitated complex were examined by qRT-PCR as described above.

### 4.9. Western Blotting

Whole-cell lysates were prepared with Mammalian Protein Extract Reagent (M-PER; Thermo Scientific, Rockford, IL, USA). Cells in a 6-well culture plate were washed with PBS and 200 μL of M-PER was then added. The whole-cell lysates were transferred into a 1.5 mL tube and sonicated. The proteins (cell lysate and exosomes) denatured in 4 × sample buffer solution without 2-Mercaptoethanol (Wako Pure Chemical Industries, Tokyo, Japan) were loaded onto a Mini- PROTEAN TGX Gel (4–20%, Bio-Rad) and electrotransferred (100 V, 30 mA) for 1 h. The proteins were transferred to a 0.45 µm polyvinylidene difluoride membrane (Millipore, Merck Sigma, MA, USA). After blocking in Blocking One (Nacalai Tesque, Kyoto, Japan) for 1 h at 25 °C, the membranes were incubated overnight at 4 °C with primary antibodies, which included anti-CD63 (purified mouse anti-human CD63, H5C6, 556019, BD, dilution 1:200), anti-CD9 (ALB6, sc-59140, Santa Cruz Biotechnology Inc., Dallas, TX, USA, dilution 1:200), anti-CD81 (Santa Cruz Biotechnology Inc., dilution 1:200), anti-actin (clone C4, MAB1501, Millipore, dilution 1:1000) and anti-CD274 (E1L3N XP Rabbit mAb, 13684, Cell Signaling, dilution 1:1000) or GAPDH (MAB374, Millipore, dilution 1:1000) antibodies. The secondary antibodies (horseradish peroxidase-linked anti-mouse IgG, NA931 or horseradish peroxidase-linked anti-rabbit IgG, NA934, GE Healthcare) were used at a dilution of 1:5000. The membrane was then exposed to ImmunoStar LD (Wako, Osaka, Japan). The full membranes of Western blotting were attached in Supplementary Figure S1.

### 4.10. Tumor Cell/T Cell Coculture Model

Peripheral blood mononuclear cells (PBMCs) or T cells isolated from PBMCs using the EasySep Human T Cell Isolation Kit (STEMCELL, STEMCELL Technologies Inc., Vancouver, Canada) were plated at a density of 1 × 10^6^ per mL in six-well plates and stimulated with anti-CD3 and CD28 antibodies for 48 h to promote T cell activation according to the T cell activation protocol provided by STEMCELL (https://www.stemcell.com/immunocult-human-cd3-cd28-cd2-t-cell-activator.html). MM231 cells were incubated with EVs-424 or EVs for 32 h. The stimulated PBMCs were cocultured with the tumor cells at a ratio of 10:1 for 16 h. The stimulated T cells were harvested and cocultured with the tumor cells at a ratio of 1:1 for 16 h. The medium from the PBMCs and tumor cell cocultures was assayed for IFN-γ, TNF-α, IL-6 and IL-10 using a cytokine enzyme-linked immunosorbent assay (ELISA).

### 4.11. Immunofluorescence

For internalization of exosomes derived from AT-MSCs, EVs were labeled with a PKH26 red fluorescence labeling kit according to the manufacturer’s instruction (Sigma-Aldrich, Thermo Fisher Scientific, Waltham, MA, USA). Briefly, 30 µg of exosomes in 90 µL of PBS were mixed with 360 µL of 8 µM PKH26 and incubated for 5 min with mixing by gentle pipetting. The excess dye was absorbed with 360 µL of 1% BSA in PBS. Then, the EVs were washed with PBS at 110,000× *g* for 70 min. The pellets were resuspended in 500 µL of PBS and transferred into a 100-kDa filter (Microcon YM-100, Millipore, Merck Sigma, MA, USA), and the cell suspension was concentrated to 30 µL. PKH26-labeled EVs were added to the recipient cells. After 24 h of incubation, images were acquired using confocal microscopy (FV10i; Olympus Life Science, Tokyo, Japan).

### 4.12. Apoptosis Assessment of Tumor Cells in the Coculture System

MM231 were cocultured with stimulated T cells for 16 h after transfection with miR-424-5p mimics for 24 h or incubation with EVs-424 or EVs for 32 h. Subsequently, the apoptosis of MM231 cells were evaluated using two methods, including the caspase activity of the coculture system and lactate dehydrogenase (LDH)-release assay.

### 4.13. Caspase 3/7 Assay

The measurements of caspase activity in the tumor cell/T cell coculture system were performed using the commercially available Caspase-Glo 3/7 assay (Promega, Madison, WI, USA) according to the manufacturer’s instructions. Briefly, after coculture, 100 µL of Caspase-Glo 3/7 reagent (Promega, Promega Corporation, Madison, WS, USA) was added to each well. The plates were shaken at 400 rpm for 3 min and incubated in the incubator for 1.5 h. The luminescence was recorded using a microplate reader (SpectraMax iD5, Molecular Devices, CA, USA).

### 4.14. LDH Release Assay

After coculture, the intracellular content release was measured using the Cytotoxicity LDH Assay Kit-WST (Dojindo Laboratories, Kumamoto, Japan) according to the manufacturer’s protocol. The absorbance was recorded at 490 nm using a microplate reader. Apoptosis of tumor cells was calculated following the formula: % cell lysis = (experimental − T spontaneous − low control) × 100/(high control-low control), where “experimental” corresponds to the signal value of all cells in the coculture system, “T spontaneous” to the spontaneous background signal value of the T cells alone, “low control” to the signal value of tumor cells alone and “high control” to the maximum signal value of tumor cells with lysis buffer.

### 4.15. In Vivo Studies

The model was slightly modified according to previous studies [54]. All animal studies were performed in compliance with the guidelines of the Institute for Laboratory Animal Research, National Cancer Center Research Institute (Number: T19-010, 11 June 2019). Five-week-old female Balb/c athymic nude mice (CLEA Japan, Shizuoka, Japan) were anesthetized by exposure to 3% isoflurane via injection and subjected to in vivo imaging. MDA-MB-231-D3H2LN cells (2 × 10^6^) and 1 × 10^6^ stimulated PBMCs in 50 µL of PBS were mixed with 50 µL of Matrigel (Corning, Merck Sigma, MA, USA), and then injected subcutaneously into the fat pad of the mice. Two days later, 30 µg of Exo-424 or Exo, in 100 µL of PBS was injected intratumorally every other day 4 times in total. The tumor mass was evaluated by measuring the bioluminescence with the IVIS Spectrum (Xenogen, Caliper, New York, USA) according to the manufacturer’s instructions. The data were analyzed using LIVINGIMAGE software (version 2.50, Xenogen, Caliper, New York, NY, USA).

### 4.16. Statistics

Data were expressed as means ± standard deviation (SD) (*n* > 3). Multiple group comparisons were evaluated via a one-way analysis of variance (ANOVA) followed by a least significant difference *t*-test for post hoc analysis. Data between two independent groups were compared using a two-tailed Student’s *t*-test. Analyses were performed using SPSS software (Chicago, IL, USA). *p* < 0.05 was considered to represent a significant difference.

## Data Availability

Data sharing not applicable.

## Supplementary Materials

The following are available online at https://www.mdpi.com/1422-0067/22/2/844/s1.
