# Supplementary material for: Delivery of miR-424-5p via Extracellular Vesicles Promotes the Apoptosis of MDA-MB-231 TNBC Cells in the Tumor Microenvironment
Source: Int J Mol Sci. 2021 Jan 15;22(2):844. doi: 10.3390/ijms22020844 (PMC7831022; doi:10.3390/ijms22020844)
Supplement: Supplementary file 1 [file ijms-22-00844-s001.zip › Supplementary Tables.docx]

Supplementary. Table S1A. Correlations between PD-L1 expression and clinicopathological factors in TNBC.

| Characteristics |  | Number | High (%) | Low (%) | P |
| --- | --- | --- | --- | --- | --- |
| Age (years) | ≤45 | 38 | 12(31.58) | 26(68.42) | 0.027 |
|  | ＞45 | 176 | 95(53.98) | 81(46.02) |  |
| Grade | I | 25 | 16(64.00) | 9(36.00) | 0.317 |
|  | II | 143 | 70(48.95) | 73(51.05) |  |
|  | III, IV | 46 | 21(45.65) | 25(54.35) |  |
| Status | Dead | 165 | 86(52.12) | 79(47.88) | 0.038 |
|  | Alive | 49 | 21(42.86) | 28(57.14) |  |

Table S1B. Correlations between PD-L1 expression and clinicopathological factors in non-TNBC.

| Characteristics |  | Number | High (%) | Low (%) | P |
| --- | --- | --- | --- | --- | --- |
| Age (years) | ≤45 | 95 | 54(56.84) | 41(43.16) | 0.208 |
|  | ＞45 | 517 | 252(48.74) | 265(51.26) |  |
| Grade | I | 118 | 57(48.31) | 61(51.69) | 0.140 |
|  | II | 324 | 174(53.70) | 150(46.30) |  |
|  | III, IV | 170 | 75(44.12) | 95(55.88) |  |
| Status | Dead | 513 | 262(51.07) | 251(48.93) | 0.425 |
|  | Alive | 99 | 44(44.44) | 55(55.56) |  |
