# Supplementary figures and images for: Delivery of miR-424-5p via Extracellular Vesicles Promotes the Apoptosis of MDA-MB-231 TNBC Cells in the Tumor Microenvironment
Source: Int J Mol Sci. 2021 Jan 15;22(2):844. doi: 10.3390/ijms22020844 (PMC7831022; doi:10.3390/ijms22020844)

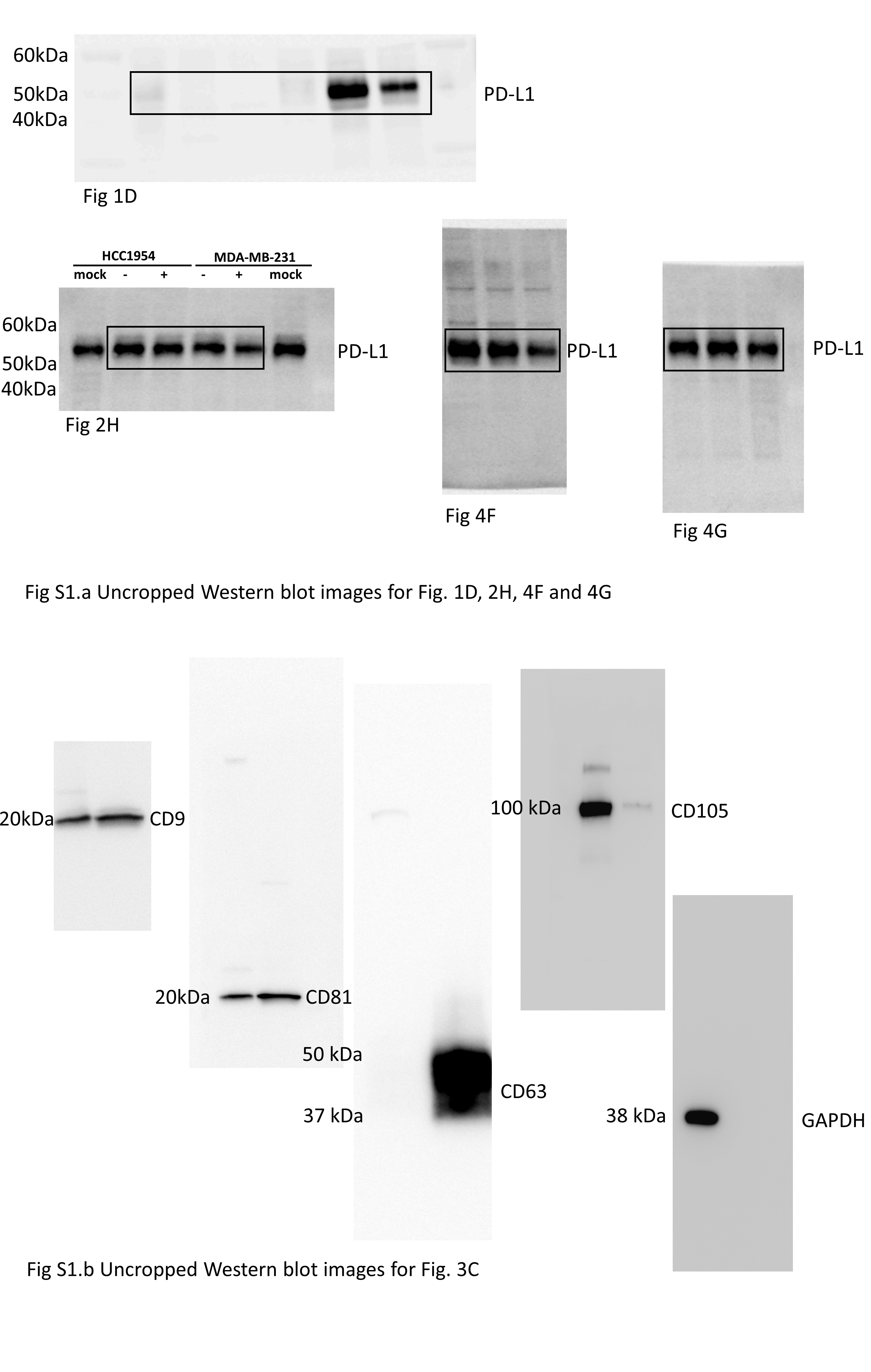

Supplement: Supplementary file 1 [file ijms-22-00844-s001.zip › Supplementary Figure S1.tif]
